# Editorial: Computational modelling of cardiovascular hemodynamics and machine learning

**DOI:** 10.3389/fcvm.2024.1355843

**Published:** 2024-02-22

**Authors:** Christos Bourantas, Ryo Torii, Sergey Karabasov, Rob Krams

**Affiliations:** ^1^ Department of Cardiology, Bart’s Heart Centre, Barts Health NHS Trust, London, United Kingdom; ^2^ Device and Innovation Centre, William Harvey Research Institute, Queen Mary University, London, United Kingdom; ^3^ Department of Mechanical Engineering, University College, London, United Kingdom; ^4^ School for Science and Engineering, Queen Mary University, London, United Kingdom

**Keywords:** artificial intelligence, machine learning, shear stress, imaging, navier stokes solvers

**Editorial on the Research Topic** Computational modelling of cardiovascular hemodynamics and machine learning

Artificial Intelligence (A.I.) holds promises in many fields, especially in the health sector. Here, pattern recognition of complex problems—a major strength of A.I. is what makes A.I. so useful. Despite its promises, applying A.I. to the health sector comes with specific challenges, of which the papers in the current issue aim to provide solutions (1–3). This editorial will offer specific background theory on A.I. to better understand the solutions offered in this issue.

The general requirements for high precision, generalizable A.I. models are the data set on which the model is trained/validated is homogeneous, well-labelled (for supervised learning), the data sets are of sufficient size, and the data set contains more measurements repeats than features (−10:1) (4, 5). The size of the data set is dependent on the complexity of the model. In general, the rule of thumb is to have 10 times as much data as the number of parameters in your model. As a deep neural network (NN) contains easily more than 100,000 parameters, data sets of 1M points (e.g., 1M patients) are often required, which is quite an unrealistic assumption. Hence, several engineering methods have been proposed to overcome this challenge, like data augmentation, transfer learning or data synthesis. Some of these methods have been proposed in the current issue.

The neural network predictions are best described by conditional Bayesian statistics, which depend not only on the statistical distributions (variance, mean, outliers, missing values) of the data sets (e.g., feature space), but also on the modelling complexity (e.g., weight distribution) (6–8). There is a well-known trade-off between variance and bias which is determined by the modelling complexity and size of the feature space (6–8). Increasing complexity of the model at constant input, reduces the variance and improves training predictions but tend to increase the bias thereby reducing the generalizability of the model to new data sets. A recent study shed light on how model generalizability is affected. The two main factors are the curvature of the loss function, and the variability of the weights within each layer of the network (9). Well-known procedures to improve generalizability (tuning parameters, regularisation, drop out) affected these two parameters as expected (10, 11). It is therefore essential to not only validate the model by splitting the data set in training and validation data, but also to test the model on new, independent data. Prediction accuracies need to be high in both the validation and testing studies.

This issue of Frontiers had as subject Computational modelling, Cardiovascular Hemodynamic and Artificial Intelligence and contained 9 papers. Two (2) papers in this issue focussed on rapid flow modelling with A.I. In the first paper (Pavlo Yevtushenko et al.), a RNN (1,100 nodes) was trained on 244 patients and was validated on 23 patients using stationary flow data generated with Computational Fluid Dynamics (CFD) applied on a systolic geometry of aortic valves and the proximal aorta obtained with a combination of echocardiography and computed tomography (CT) imaging. The NN predicted the pressure and wall shear stress distribution over the centreline geometry with >90% accuracy and within seconds. The limitations of this approach are: (i) the simplicity of the ground truth (stationary flow, stiff walls, absence of moving valves), (ii) relatively small data set and (iii) the absence of testing on an independent data set. The second study (Morgan et al.) used the CFD derived ground truth but predicted the velocity field instead of pressure and shear stress. This creates a higher flexibility to predict more sophisticated biomechanical factors derived from the velocity field. The training set was created using synthetic data generation algorithm, a novel method to overcome the “curse of dimensionality” of A.I. In addition, it was optimised for speed, by using state-of-the art feature reduction methods (t-SNE) and Proper Orthogonal Decomposition of the velocity field (cPOD). This method could easily be extended to include time-dependence, elastic walls, and moving boundaries, making this method applicable to a variety of health problems.

Non-invasive technologies (Computer Tomography (CT), magnetic resonance imaging (MRI)) have been increasingly used for screening in Cardiology. These techniques are getting more sophisticated in their speed, and analysis, but have a lower spatial resolution (−200 micron) than invasive techniques (−10–50 micron). In addition, the radiation dose of CT is high, preventing the creation of time series. In two review papers, the authors (Liao et al. and Baeßler et al.) describe A.I. studies aimed at reducing imaging noise when radiation dose in CT is reduced. It is in this application, that CNN models proved to be superior to more classical methods (regression trees, support vector machine, k-NN). The same studies indicated that A.I. methods are useful for vessel segmentation, and stenosis detection. While these studies indicate a high sensitivity of DNN classifiers, the specificity of these Deep Neural Network models appeared rather low. This is probably due to the effect of noise in the feature space on classification, as indicated above. AI for calcification classification and plaque composition detection with CT radiometry shows improvement over the more classical methods but needs further development because of low accuracies. In their review papers, Liao and Baessler discussed coronary artery stenosis detection and fractional flow reserve (FFR). Their review indicated that both can be detected with high accuracy.

Two studies investigated the origins of errors in parameters derived from flow measurements in models of idealised coronary stenosis (Nguyen Ho et al.), and cardiac output derived from CT coronary aniography (Leiknes et al.). These studies indicate that the major problem is in classification of the anatomical parameters. In order to overcome this problem, Nguyen et al. applied ensemble averaging. The principle is based on repeated analysis of slightly different model parameters, thereby reducing Bayesian error propagation in neural networks. Indeed, this technique showed an improvement of classification accuracy over classical backpropagation methods and feature averaging methods.

In summary, A.I. models are increasingly applied to problems in Cardiology. These models show excellent results in detecting the complicated patterns underlying cardiovascular disease. The requirement to acquire a well-validated, generalizable predictor are demanding and are briefly discussed in the introduction of this paper. As most models presented here use rather small data sets, their accuracy and generalizability may be improved. Besides increasing the feature space (number of cases), several solutions for working with small data sets have been introduced in this journal, like ensemble averaging, and synthetic data generation.
